# Embolização para veia cava inferior de cateter totalmente implantável para quimioterapia

**DOI:** 10.1590/1677-5449.007717

**Published:** 2018

**Authors:** Barhbara Brenda Dias Garcez, Walberto Monteiro Neiva Eulálio, Sabas Carlos Vieira

**Affiliations:** 1 Universidade Estadual do Piauí – UESPI, Faculdade de Medicina, Teresina, PI, Brasil.; 2 Universidade Federal do Piauí – UFPI, Faculdade de Medicina, Teresina, PI, Brasil.

**Keywords:** cateteres, cateteres de demora, complicações pós-operatórias, veias cavas

## Abstract

A fratura com embolização de cateter inserido perifericamente em pacientes que receberam quimioterapia representa uma complicação grave e rara, constituindo menos de 1% das complicações relacionadas a esse procedimento. Relatamos aqui um caso de embolização de cateter totalmente implantável em uma paciente de 57 anos submetida a laparotomia por lesão anexial complexa devido a um câncer de ovário com carcinomatose intraperitoneal disseminada diagnosticado no intraoperatório. A paciente foi submetida a histerectomia e salpingooforectomia bilateral, não sendo realizada cirurgia oncológica radical. A análise histopatológica revelou adenocarcinoma de ovário G3. Em outubro de 2013, exame radiológico de rotina diagnosticou fratura e embolização de segmento distal do cateter para veia cava inferior retro e supra-hepática. A paciente não apresentou nenhuma sintomatologia. Procedeu-se à retirada do cateter através da veia femoral pela técnica do laço, sem complicações. Paciente está sem evidência de doença 24 meses após a realização do procedimento.

## INTRODUÇÃO

A fratura com embolização de cateter totalmente implantável para quimioterapia representa menos de 1% das complicações relacionadas a esse dispositivo. Ela ocorre por compressão do cateter pela primeira costela e clavícula, conhecido como síndrome de *pinch-off*. A embolização do fragmento pode ocorrer em átrio, ventrículo, artéria pulmonar e veia cava^1^.

Utilizando os termos *embolization*, *catheter*, *chemotherapy* e *fracture* na base de dados PubMed, foi encontrado apenas um caso de fratura e embolização de cateter para quimioterapia para a veia cava inferior^2^, justificando a apresentação do presente caso.

### Parte I – Situação clínica

Paciente feminina, 57 anos de idade, submeteu-se em janeiro de 2010 a laparotomia por lesão anexial complexa, sendo diagnosticado no intraoperatório câncer de ovário com carcinomatose peritoneal disseminada, sendo realizada somente histerectomia e salpingooforectomia bilateral, não sendo realizada cirurgia oncológica radical. O exame histopatológico revelou adenocarcinoma de ovário G3. Procedeu-se em fevereiro de 2010 à instalação por punção de um cateter totalmente implantável para quimioterapia na veia subclávia direita, cujo procedimento ocorreu sem intercorrências. O segmento distal do cateter foi deixado na veia cava superior, próximo à entrada no átrio direito.

**Figura 1:**
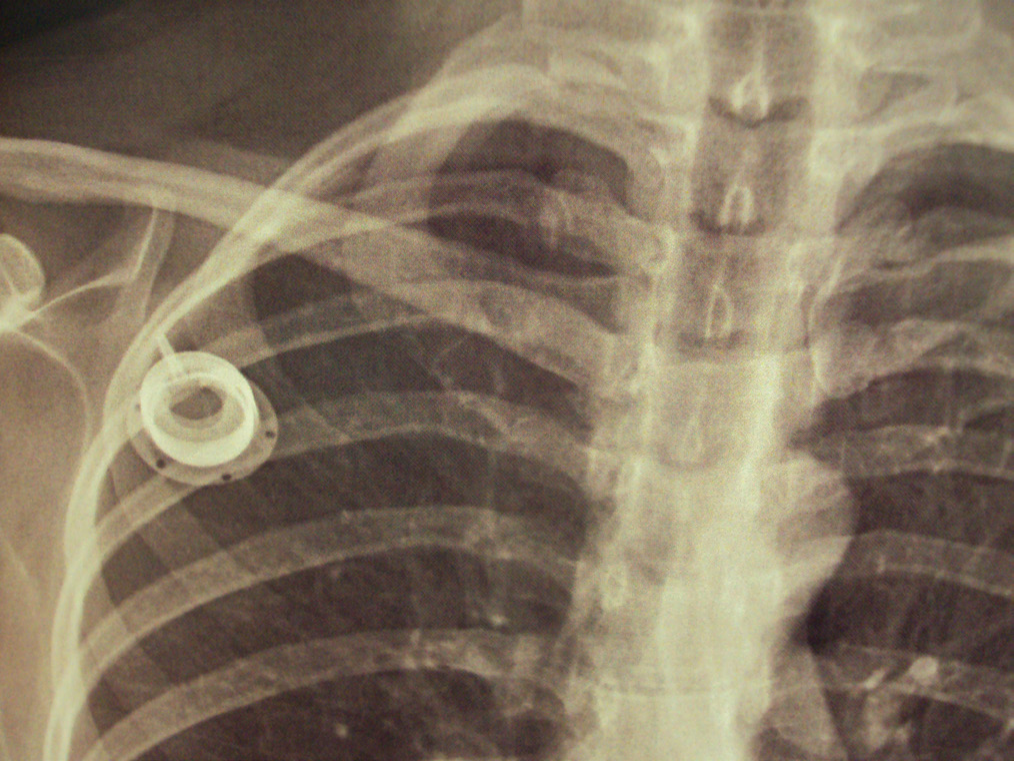
Raio X de tórax mostrando o reservatório sem o cateter.

**Figura 2:**
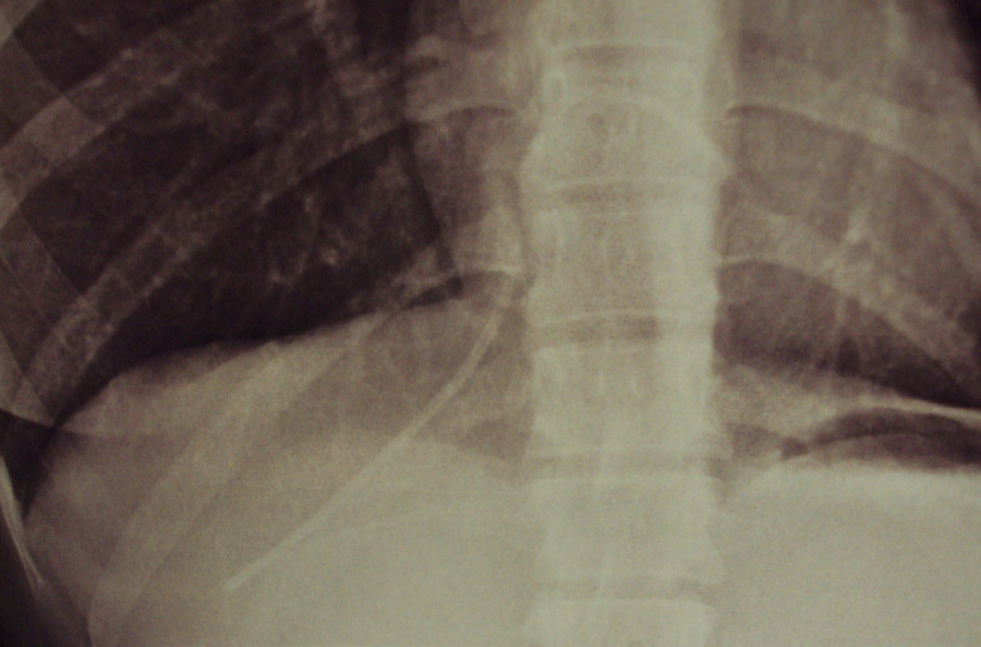
Raio X de tórax demonstrando o cateter fraturado e embolizado para veia cava inferior.

A paciente submeteu-se a quimioterapia adjuvante baseada em platina e taxol, apresentando boa tolerância. Entretanto, em outubro de 2013, exame radiológico de rotina diagnosticou fratura (Figura 1) e embolização de segmento distal do cateter para veia cava inferior retro e supra-hepática (Figura 2). A paciente não apresentou nenhuma sintomatologia.

### Parte II – O que foi feito

**Figura 3:**
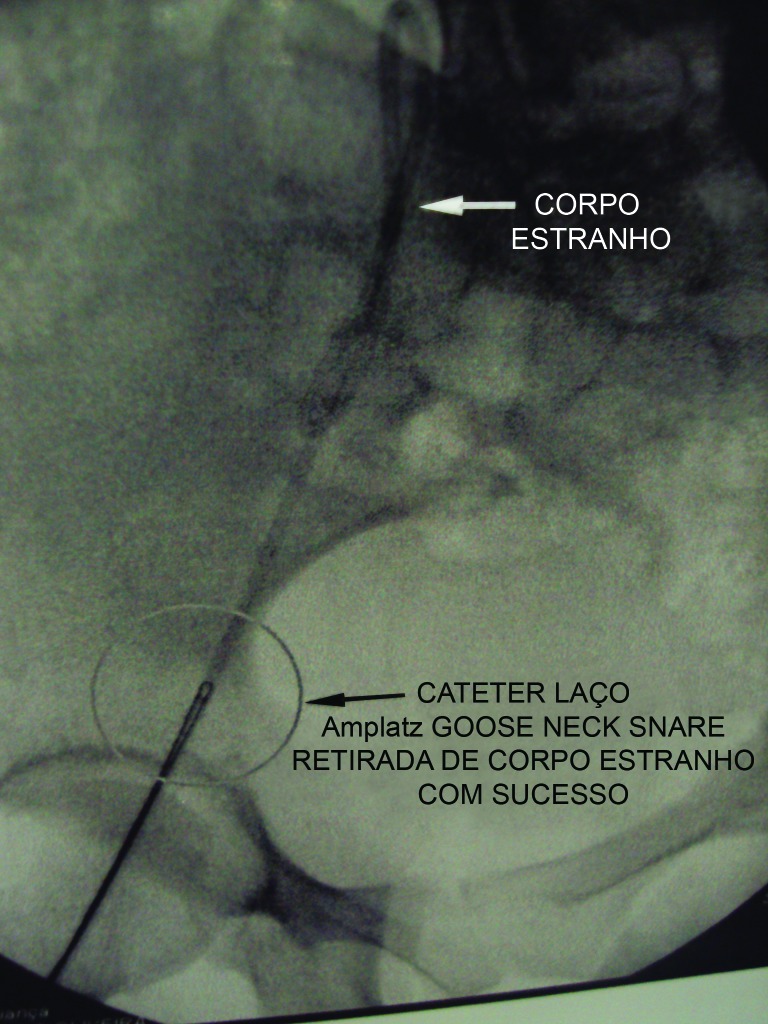
Recuperação do fragmento embolizado pela técnica do laço.

Após preparo pré-operatório, a paciente foi encaminhada para o centro cirúrgico. Sob anestesia geral, procedeu-se à retirada do cateter através da veia femoral pela técnica do laço (Figura 3). O procedimento ocorreu sem complicações. Em seguida, o reservatório foi removido. A paciente evoluiu bem, recebendo alta hospitalar no dia seguinte. Atualmente, está sem evidência de doença oncológica 24 meses após a realização do procedimento.

## DISCUSSÃO

O uso de cateter totalmente implantável normalmente é indicado para pacientes que necessitam de quimioterapia de longa duração para neoplasias malignas^1^. Seu uso é descrito com baixa taxa de complicação^3^^,^ 4. Entretanto, a permanência prolongada pode levar a uma série de complicações, uma delas potencialmente letal: a fratura e embolização de fragmentos do cateter. Esse é um evento raro, que corresponde a cerca de 1% das complicações relacionadas a esse dispositivo^5^. Os locais mais frequentes de embolização são para átrio, ventrículo e artéria pulmonar. Os fragmentos de cateter podem causar complicações como perfuração cardíaca, arritmias, sepse e embolia pulmonar, por se comportarem como um corpo estranho no sistema venoso^6^.

O mecanismo da fratura ocorre pela compressão do cateter na passagem entre a clavícula e a primeira costela, levando ao estresse do material, podendo ocorrer a ruptura parcial ou total. A ocorrência da fratura ocorre geralmente quando o cateter é colocado por punção. O implante, quando realizado por dissecção, seja da veia cefálica ou jugular externa, apresenta uma taxa de fratura menor, pois o cateter não passa entre a primeira costela e a clavícula^6^.

No presente caso, a fratura ocorreu 26 meses depois da instalação do cateter. Após o término da quimioterapia, se o paciente tem bom prognóstico, o dispositivo deve ser retirado prontamente. No entanto, para pacientes com prognóstico reservado, como câncer de ovário avançado, em que a taxa de recidiva é alta, é prudente deixar o cateter, devido à possibilidade da paciente necessitar de quimioterapia se ocorrer recidiva. A embolização ocorreu para veia cava inferior retro e supra-hepática. Até onde temos conhecimento, apenas um caso de fratura e embolização de cateter para veias hepáticas foi descrito na literatura^2^.

Geralmente a embolização é assintomática, sendo diagnosticada quando da punção para infusão, coleta de sangue ou heparinização, não ocorrendo refluxo de sangue, o que deve chamar a atenção para a possibilidade de embolização. Nesses casos, uma radiografia simples de tórax estabelece o diagnóstico. A ocorrência de óbito por embolização de cateter totalmente implantável para quimioterapia é um evento raro^7^.

Os sinais e sintomas associados à síndrome de pinch-off envolvem dificuldade na infusão de fluidos na posição de repouso, tendo o paciente que abduzir seu membro superior a fim de ampliar o ângulo costoclavicular e eliminar a compressão do cateter. O diagnóstico é feito pela análise radiográfica simples de tórax com visualização do reservatório desconectado da parte distal^8^. O tratamento deve ser realizado o mais precocemente possível, sendo a técnica por acesso endovascular o tratamento padrão, por apresentar baixas taxas de complicações^9^, como no presente relato, em que o procedimento ocorreu sem intercorrências.

A partir disso, percebe-se que a embolização para veia cava inferior de fragmentos de cateter venoso central totalmente implantável é uma complicação extremamente rara e potencialmente letal. A equipe deve ficar atenta a qualquer sinal de dificuldade de coleta de sangue ou administração de líquidos. O diagnóstico pode ser feito por meio de radiografia simples e o tratamento de eleição é a retirada por abordagem endovascular.
