# Supplementary material for: Embolização para veia cava inferior de cateter totalmente implantável para quimioterapia
Source: J Vasc Bras. 2018 Jan-Mar;17(1):89–92. [Article in Portuguese] doi: 10.1590/1677-5449.007717 (PMC5990261; doi:10.1590/1677-5449.007717)
Supplement: Translation

# Translation: Totally implantable chemotherapy catheter embolization in inferior vena cava

**Authors:** Barhbara Brenda Dias Garcez, Walberto Monteiro Neiva Eulálio, Sabas Carlos Vieira

**Affiliations:** 1 Universidade Estadual do Piauí – UESPI, Faculdade de Medicina, Teresina, PI, Brasil.; 2 Universidade Federal do Piauí – UFPI, Faculdade de Medicina, Teresina, PI, Brasil.

**Keywords:** catheters, catheters, indwelling, postoperative complications, vena cava

## Abstract

Fracture of a peripherally inserted catheter causing embolization in patients on chemotherapy is a serious and rare complication, constituting less than 1% of complications related to this procedure. We report here a case of fully implantable catheter embolization in a 57-year-old female who had undergone laparotomy for complex adnexal lesion due to ovary cancer with disseminated peritoneal carcinomatosis, diagnosed intraoperatively. The patient was treated with hysterectomy and bilateral salpingo-oophorectomy, and radical oncological surgery was not performed. Histopathological analysis revealed G3 ovarian adenocarcinoma. In October 2013, a routine radiological examination diagnosed fracture and embolization of the distal segment of the catheter into the retrohepatic and suprahepatica inferior vena cava. The patient did not present any symptoms. The catheter was withdrawn through the femoral vein using the snare technique, without complications. The patient has no evidence of disease 24 months after the procedure.

## INTRODUCTION

Fracture of a totally implantable chemotherapy catheter followed by embolization accounts for less than 1% of the complications related to use of this device. It is caused by compression of the catheter by the first rib and clavicle, which is known as pinch-off syndrome. Embolization of the fragment can occur in the atrium, ventricle, pulmonary artery, or vena cava.^1^

A search of the PubMed database using the keywords embolization, catheter, chemotherapy, and fracture returned just one report of a case of fracture of a chemotherapy catheter with embolization in the inferior vena cava,^2^ demonstrating the relevance of presenting this case.

### Part I – Clinical situation

In January 2010, a 57-year-old female patient underwent laparotomy to treat a complex adnexal lesion and was diagnosed intraoperatively with ovarian cancer with disseminated peritoneal carcinomatosis, which was managed with hysterectomy and bilateral salpingo-oophorectomy, without radical oncological surgery. Histopathological examination revealed G3 ovarian adenocarcinoma. In February 2010, a totally implantable chemotherapy catheter was fitted by puncture into the right subclavian vein. The procedure was conducted with no complications. The distal segment of the catheter was placed in the superior vena cava, close to the entrance to the right atrium.

The patient was treated with adjuvant chemotherapy, based on platina and taxol, which she tolerated well. However, in October 2013, a routine radiological examination diagnosed fracture (Figure 1) and embolization of the distal segment of the catheter into the retrohepatic and suprahepatic inferior vena cava (Figure 2). The patient did not exhibit any symptoms.

**Figure 1:** Chest X-ray showing the port without the catheter.

**Figure 2:** Chest X-ray showing the fractured catheter embolized in the inferior vena cava.

### Part II – What was done

After preoperative preparation, the patient was transferred to the operating theater. Under general anesthesia, the catheter was withdrawn via the femoral vein, using the snare technique (Figure 3). The procedure was conducted with no complications. The port was then removed. The patient recovered well and was discharged from hospital the next day. She is currently free from any evidence of cancer, 24 months after the procedure.

**Figure 3:** Recovery of the embolized fragment using the snare technique.

## DISCUSSION

Totally implantable catheters are normally indicated for patients who require long-duration chemotherapy for malignant neoplasms.^1^ Their use is associated with a low rate of complications.^3^^,^ 4 However, if they remain in place for prolonged periods, a series of complications are possible, one of which is potentially lethal: fracture and embolization of catheter fragments. This is a rare event, accounting for around 1% of the complications related to this device.^5^ The most common embolization sites are the atrium, ventricle, and pulmonary artery. Catheter fragments can cause complications such as cardiac perforation, arrhythmia, sepsis, and pulmonary embolism, since they behave like a foreign body in the venous system.^6^

The mechanism of fracture is by compression of the catheter between the clavicle and the first rib, putting stress on the material, and rupture can be partial or total. Fractures generally occur when the catheter has been placed by puncture. Implantation by dissection, whether of the cephalic or external jugular vein, is associated with a lower rate of fracture because the catheter does not pass between the first rib and the clavicle.^6^

In the present case, fracture occurred 26 months after the catheter was fitted. After chemotherapy has been completed, if the patient’s prognosis is good, the device should be removed promptly. However, for patients with poorer prognosis, such as those with advanced ovarian cancer, which has a high rate of relapse, it is prudent to leave the catheter in place because of the possibility that the patient may need chemotherapy if a relapse takes place. Embolization was into the retrohepatic and suprahepatic inferior vena cava. To our knowledge, only one case of fracture and embolization of a catheter in hepatic veins has been described in the literature.^2^

Embolization is generally asymptomatic and is diagnosed when the port is punctured for infusion, to draw blood, or for heparinization and reflux of blood is absent, which should call attention to the possibility of embolization. In these cases, a simple chest X-ray can confirm diagnosis. Death caused by embolization of a totally implantable chemotherapy catheter is a rare event.^7^

The signs and symptoms associated with pinch-off syndrome involve difficulty infusing fluids with the patient at rest, requiring the patient to abduct the upper limb in order to widen the costoclavicular angle and eliminate compression of the catheter. Diagnosis is made by analysis of simple chest X-rays showing the port detached from the distal part.^8^ Treatment should be provided as early as possible and the endovascular access technique is the standard treatment, because it is associated with low rates of complications,^9^ as in the case described here, in which the procedure was conducted with no intercurrent complications.

Embolization to the inferior vena cava of fragments of a totally implantable central venous catheter is an extremely rare and potentially lethal complication. The treating team should be alert to any sign of difficulty drawing blood or administering liquids. Diagnosis can be made by simple X-ray and the treatment of choice is removal via an endovascular approach.
